# Intestinal Immunity to Poliovirus Following Sequential Trivalent Inactivated Polio Vaccine/Bivalent Oral Polio Vaccine and Trivalent Inactivated Polio Vaccine–only Immunization Schedules: Analysis of an Open-label, Randomized, Controlled Trial in Chilean Infants

**DOI:** 10.1093/cid/ciy603

**Published:** 2018-10-30

**Authors:** Elizabeth B Brickley, Wendy Wieland-Alter, Ruth I Connor, Margaret E Ackerman, Austin W Boesch, Minetaro Arita, William C Weldon, Miguel G O’Ryan, Ananda S Bandyopadhyay, Peter F Wright

**Affiliations:** 1 Department of Epidemiology, Geisel School of Medicine, Dartmouth College, Hanover, New Hampshire; 2 Department of Infectious Disease Epidemiology, London School of Hygiene & Tropical Medicine, United Kingdom; 3 Department of Pediatrics, Dartmouth-Hitchcock Medical Center, Lebanon; 4 Department of Microbiology and Immunology, Geisel School of Medicine, Dartmouth College, Hanover, New Hampshire; 5 Thayer School of Engineering, Dartmouth College, Hanover, New Hampshire; 6 Department of Virology II, National Institute of Infectious Diseases, Tokyo, Japan; 7 Division of Viral Diseases, Centers for Disease Control and Prevention, Atlanta, Georgia; 8 Microbiology and Mycology Program and Millennium Institute of Immunology and Immunotherapy, Faculty of Medicine, University of Chile, Santiago; 9 Bill & Melinda Gates Foundation, Seattle, Washington

**Keywords:** poliovirus, inactivated vaccine, live oral vaccine, human challenge, mucosal immunity

## Abstract

**Background:**

Identifying polio vaccine regimens that can elicit robust intestinal mucosal immunity and interrupt viral transmission is a key priority of the polio endgame.

**Methods:**

In a 2013 Chilean clinical trial (NCT01841671) of trivalent inactivated polio vaccine (IPV) and bivalent oral polio vaccine (bOPV; targeting types 1 and 3), infants were randomized to receive IPV-bOPV-bOPV, IPV-IPV-bOPV, or IPV-IPV-IPV at 8, 16, and 24 weeks of age and challenged with monovalent oral polio vaccine type 2 (mOPV2) at 28 weeks. Using fecal samples collected from 152 participants, we investigated the extent to which IPV-bOPV and IPV-only immunization schedules induced intestinal neutralizing activity and immunoglobulin A against polio types 1 and 2.

**Results:**

Overall, 37% of infants in the IPV-bOPV groups and 26% in the IPV-only arm had detectable type 2–specific stool neutralization after the primary vaccine series. In contrast, 1 challenge dose of mOPV2 induced brisk intestinal immune responses in all vaccine groups, and significant rises in type 2–specific stool neutralization titers (*P* < .0001) and immunoglobulin A concentrations (*P* < 0.0001) were measured 2 weeks after the challenge. In subsidiary analyses, duration of breastfeeding also appeared to be associated with the magnitude of polio-specific mucosal immune parameters measured in infant fecal samples.

**Conclusions:**

Taken together, these results underscore the concept that mucosal and systemic immune responses to polio are separate in their induction, functionality, and potential impacts on transmission and, specifically, provide evidence that primary vaccine regimens lacking homologous live vaccine components are likely to induce only modest, type-specific intestinal immunity.

The Global Polio Eradication Initiative successfully completed a worldwide replacement of the trivalent oral polio vaccine (tOPV) with a bivalent types 1 and 3 oral polio vaccine (bOPV) in spring 2016 [1]. The withdrawal of live serotype 2 poliovirus from the oral polio vaccines (OPVs) delivered in routine immunization programs was coupled with country-level commitments to introduce at least 1 dose of the trivalent inactivated polio vaccine (IPV) [2]. This global switch represents an important advance for efforts to prevent vaccine-associated paralytic poliomyelitis cases [3] and to mitigate risks of introducing new circulating vaccine-derived polioviruses from the type 2 component of the oral vaccine [4]. Nevertheless, the implications of IPV-only and IPV-bOPV immunization schedules for the acquisition of mucosal immunity to polio remain uncertain.

In a similar way to how vaccine-induced systemic immunity is important for the prevention of paralytic poliomyelitis, vaccine-induced mucosal immunity is critical for the interruption of poliovirus transmission and, therefore, of paramount importance in the journey towards global polio eradication [5, 6]. Although IPV-driven immunization schedules have successfully eliminated polio from high-income regions via improved sanitation and hygiene practices, particularly where oral-oral rather than fecal-oral transmission predominated, such as Scandinavia and the Netherlands (reviewed in [7]), the capacity of IPV to block intestinal viral shedding upon challenge with live OPV viruses appears to be limited [8–10]. For example, in a Baltimore-based cohort, 85% of participants who had been immunized with IPV at 2, 4, and 15 months of age excreted polioviruses when challenged with tOPV at 18 months [11]. Similarly, in a Cuban cohort free of environmental exposure to OPV-derived viruses, infants immunized with 2 and 3 doses of IPV shed tOPV challenge virus at titers only modestly lower than the titers of control infants who had no prior polio experience (mean log_10_ viral titers, respectively: 3.46, 3.37, and 3.89) [12].

The present study complements this research and uses state-of-the-art immunoassays to investigate the extent to which primary immunization schedules, incorporating up to 3 doses of IPV, are capable of inducing mucosal immunity specific to polio types 1 and 2 [13]. For this open-label clinical trial, Chilean infants were randomized to receive 1 of 3 immunization schedules (ie, IPV-bOPV-bOPV, IPV-IPV-bOPV, or IPV-IPV-IPV) at 8, 16, and 24 weeks of age, followed by an mOPV2 challenge at 28 weeks. The trial’s principal findings, reported by O’Ryan et al., showed that, although all 3 vaccine regimens were associated with strong systemic responses against poliovirus types 1 and 3 and IPV dose-dependent systemic responses against poliovirus type 2, the vast majority of participants had detectable viral shedding in their stools 1 week after the mOPV2 challenge, regardless of their primary vaccine schedule [14]. The current results further advance our understanding of vaccine-induced mucosal immunity to poliovirus by providing new evidence that boosting serum neutralization via repeated doses of IPV does not, in turn, augment intestinal neutralizing activity or immunoglobulin A (IgA) levels. Instead, the results show that the induction of robust intestinal immunity is largely conditional on the receipt of homologous live vaccines. Finally, the evidence shows that the duration of breastfeeding may influence the magnitude of polio-specific mucosal immune parameters measured in infant fecal samples.

## MATERIALS AND METHODS

### Selection of Study Participants

**Figure 1.**
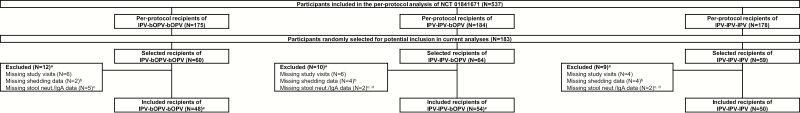
Flow diagram for the selection of the analytical cohort from NCT 01841671, an investigation of the “Immunogenicity of 1 or 2 Doses of bOPV in Chilean Infants Primed with IPV Vaccine (IPV002ABMG),” undertaken in Santiago, Chile, between 25 April and 1 August 2013. Abbreviations: bOPV, bivalent oral polio vaccine; IgA, immunoglobulin A; IPV, trivalent inactivated polio vaccine; neut., neutralization. ^a^Reasons for exclusion are not mutually exclusive. ^b^Subjects with missing type 2 oral poliovirus vaccine shedding data from at least 1 visit at 28, 29, 30, 31, or 32 weeks of age. ^c^Subjects with missing polio type 2–specific stool neutralization and immunoglobulin A data from at least 1 visit at 28 or 30 weeks of age. ^d^Polio type 1–specific immunoglobulin A data were missing from the visit at 30 weeks of age in 2 of the included subjects in the IPV-IPV-bOPV group and 1 in the IPV-IPV-IPV group. ^e^Type 1– and 2–specific serum neutralization data were missing from the visits at 28 and/or 29 weeks of age in 1 of the included subjects in the IPV-IPV-bOPV group and 3 in the IPV-bOPV-bOPV group.

The design of the phase 4, randomized, open-label, non-inferiority study (NCT01841671) undertaken between 25 April and 1 August 2013 has been previously described [14]. Participants included healthy, singleton infants aged 8 weeks (± 7 days) at the time of enrollment and excluded infants who: (1) had been previously vaccinated against poliovirus, (2) had a low birth weight (ie, <2500 g), (3) resided in a household with someone who had received OPV within the previous 6 months or was scheduled to receive OPV in the following 6 months, (4) had a confirmed or suspected immunosuppressive or immunodeficiency condition, (5) had a family history of immunodeficiency, (6) had a major congenital defect or serious chronic illness, (7) had a known allergy to any component of the vaccines, (8) had an uncontrolled blood disorder contraindicating intramuscular injections, (9) was or would be administered immunoglobulins and/or blood products since birth or during the study period, or (10) was deemed unfit for inclusion in the study based on investigator discretion. For the present analyses, 183 participants were selected at random from the 537 participants included in the per-protocol analysis of the original trial (ie, the subset of participants who received all 3 immunizations specified for the primary vaccine schedule; Figure 1). From this group of participants, an analytical cohort comprising 152 infants was selected after the exclusion of individuals who were missing mOPV2 shedding data from the 28, 29, 30, 31, or 32 week visits and/or stool neutralization/IgA data from the 28 and 30 week visits (Figure 1).

### Laboratory Procedures

Stool samples collected from the study participants at 28, 29, 30, 31, and 32 weeks of age (ie, on the day of mOPV2 challenge and at weekly intervals over the subsequent 4 weeks) were shipped frozen to the Geisel School of Medicine at Dartmouth College (Lebanon, New Hampshire) for further investigation of intestinal mucosal immunity using methods described previously [8, 9, 15, 16]. Whereas mucosal immunity to polio type 2 was the primary endpoint of interest, mucosal immunity to polio type 1 was also investigated as a comparator endpoint, reflecting that bOPV-receiving groups had exposure to a homologous live vaccine in the primary immunization series. The stool neutralization titers needed to achieve 60% neutralization were determined by limiting dilution inhibition of luciferase-labeled type-specific polio pseudoviruses in vitro, as previously described [16]: titers greater than 1:512 (ie, the highest dilution tested) were recorded at 1:1024; those less than 1:4 (ie, the lowest dilution tested) were recorded at 1:2. Total and polio type–specific concentrations of IgA in stool specimens were quantified, relative to a serum standard, using a multivariate microsphere assay developed by coupling monovalent IPVs to fluorescently-coded magnetic microspheres [15]. Vaccine group assignments were unblinded to the Dartmouth team only after sample testing and initial statistical analyses were completed.

As described in the parent study, serum neutralization titers and viral shedding were determined at the Centers for Disease Control and Prevention (Atlanta, Georgia) [14]. Type-specific serum neutralizing activity was evaluated using the World Health Organization’s standard microneutralization assay [17]. Poliovirus shedding levels were expressed as the log_10_ 50% cell culture infective dose (CCID_50_) per gram of stool. Samples with mOPV2 shedding below the limit of detection (ie, 10^2.75^ CCID_50_/mL) were recorded at a log_10_ CCID_50_ of 0. A viral shedding index was estimated, following the methods of Asturias et al. [18], as the mean of stool log_10_ type 2 viral titers at weeks 1, 2, 3, and 4 post-challenge (ie, 29, 30, 31, and 32 weeks of age). Breastfeeding data were obtained via telephone survey or directly from the clinical file; mothers were asked whether their participating infants were receiving breastmilk at 8, 16, 24, and 29 weeks of age.

### Statistical Analyses

For the statistical analyses, infants were categorized by vaccine group assignment (ie, IPV-bOPV-bOPV, IPV-IPV-bOPV, and IPV-IPV-IPV) and by reported duration of breastfeeding (ie, whether or not the child was receiving breastmilk at 29 weeks of age). Differences in the distributions of the type-specific serum and stool neutralization titers and mucosal IgA levels across vaccine regimens were evaluated using Kruskal-Wallis tests; Dunn’s tests with Bonferroni corrections estimated within each comparative trio were used to compare pairs of vaccine schedules for post-estimation hypothesis testing. Type-specific changes in the immune parameters following the mOPV2 challenge were evaluated within each of the vaccine groups using Wilcoxon matched-pairs signed ranks tests. Correlations between immune parameters were estimated using Spearman’s rank correlation coefficients and visualized by scatter plot. Longitudinal patterns in the intestinal immune response to the mOPV2 challenge were explored using boxplots in a randomly-selected subgroup of 20 infants. Differences in the proportions of infants with detectable levels of mOPV2 shedding across vaccine regimens were compared using Pearson’s chi-squared tests. Distributions of the type 2–specific stool neutralization titers and IgA levels at the 28 and 30 week study visits across breastfeeding categories were compared using Mann-Whitney U tests. Differences in the shedding index endpoint were estimated using multivariate linear regression. The association between breastfeeding status and detection of type 2–specific stool neutralization at the time of the challenge was investigated using a univariate logistic regression. All *P* values are from 2-sided statistical tests, and all analyses were performed using Stata, version 13.0 (StataCorp LP, College Station, Texas) and R, version 3.2.5.

### Ethics

The Dartmouth College Committee for the Protection of Human Subjects and local ethics committees from the Faculty of Medicine at the University of Chile, the Servicio de Salud Metropolitano Norte, and the Servicio de Salud Metropolitano Sud approved the study. The original informed consent from parents/guardians included provisions for the use of samples in future polio-related studies, so further consenting was judged unnecessary.

## RESULTS

In the present analyses, polio serotype–specific neutralizing activity and IgA concentrations were evaluated in 364 stool samples from 152 infants, representing approximately one-third of the participants in the per-protocol analysis of the primary study. In the trial, children were administered IPV-bOPV-bOPV (n = 48), IPV-IPV-bOPV (n = 54), or IPV-IPV-IPV (n = 50) at 8, 16, and 24 weeks of age and then challenged with mOPV2 at 28 weeks. Total IgA was successfully detected in all stool samples, and the median total concentration of IgA in the stool suspensions at 28 weeks of age was determined to be 94800 ng/mL (interquartile range: 45950 to 221500). The “take” of the challenge vaccine was high, as more than 95% of participants (n = 145/152) had mOPV2 shedding and/or detectable type 2–specific stool neutralizing activity over the duration of follow-up. Of note, type 2–specific viral shedding was detected during the 28-week visit in 1 infant from each of the 3 vaccine groups, suggesting that some participants in the trial were environmentally exposed to Sabin viruses prior to receipt of the live vaccine challenge.

### Effects of IPV-bOPV and IPV-only Immunization Schedules on Markers of Immunity to Polio Types 1 and 2

**Figure 2.**
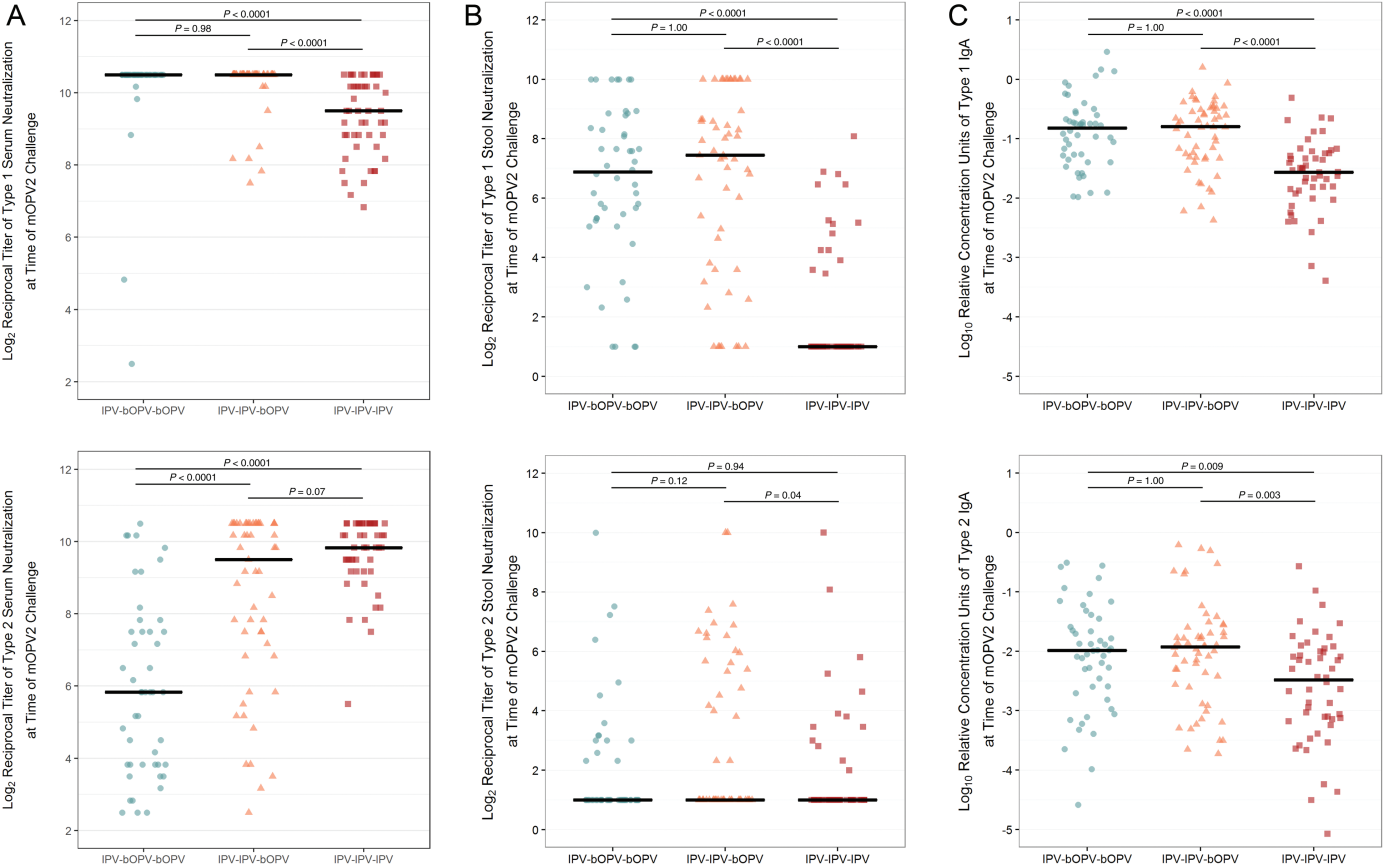
Systemic and intestinal immunity to polio types 1 and 2 at the time of the monovalent oral polio vaccine type 2 challenge (28 weeks of age) in infants previously immunized with IPV-bOPV-bOPV (n = 48), IPV-IPV-bOPV (n = 54), and IPV-IPV-IPV (n = 50). (*A*) Serum neutralization specific to poliovirus types 1 (*P* = .0001) and 2 (*P* = .0001), (*B*) stool neutralization specific to poliovirus types 1 (*P* = .0001) and 2 (*P* = .06), and (*C*) stool immunoglobulin A specific to poliovirus types 1 (*P* = .0001) and 2 (*P* = .004). Overall *P* values are from Kruskal-Wallis tests comparing the distributions of immune markers by primary vaccine group assignment; pairwise *P* values are from post hoc Dunn’s tests with Bonferroni correction for multiple testing within each grouping. Horizontal black bars indicate the median levels. Abbreviations: bOPV, bivalent oral polio vaccine; IgA, immunoglobulin A; IPV, trivalent inactivated polio vaccine; mOPV2, monovalent oral polio vaccine type 2.

Consistent with previously-reported findings [14], the primary vaccine series induced strong type 2–specific serum neutralization responses in more than 97% (n = 146/150) of the participants (Figure 2). The magnitude of serum neutralizing activity increased in an IPV dose-dependent manner, such that serum neutralization was significantly higher in the groups receiving either 2 or 3 doses of IPV compared with the group receiving a single IPV dose (Figure 2). In contrast, only 26% (n = 13/50) in the IPV-only arm and 37% (n = 38/102) of infants in the IPV-bOPV groups had detectable type 2–specific stool neutralizing activity after the primary immunization schedules (*P* = .17, Chi-squared test; Figure 2). Further, type 2–specific serum neutralization was not significantly correlated with either the relative concentration of type 2–specific stool IgA at the time of the challenge nor with the titer of peak viral shedding recorded at 1 week post-challenge (Supplementary Figure 1). An analogous disconnect between the serum and mucosal immune markers specific to polio type 1 was observed in the infants that received the IPV-only immunization schedule. While all 50 participants who received IPV alone had detectable type 1–specific serum neutralization at the time of the mOPV2 challenge, only 28% (n = 14/50) had detectable type 1–specific stool neutralization. By contrast, whereas 99% of the infants in the IPV-bOPV groups had detectable type 1–specific serum neutralization at the time of mOPV2 challenge, 89% (n = 89/100), had detectable type 1–specific stool neutralization. Additionally, infants who had received at least 1 dose of bOPV had higher reciprocal titers of type 1–specific serum neutralization and stool concentrations of type 1–specific IgA than their IPV-only counterparts (Figure 2).

### Effects of IPV-bOPV and IPV-only Immunization Schedules on Intestinal Immune Responses to Live Vaccine Challenge

**Figure 3.**
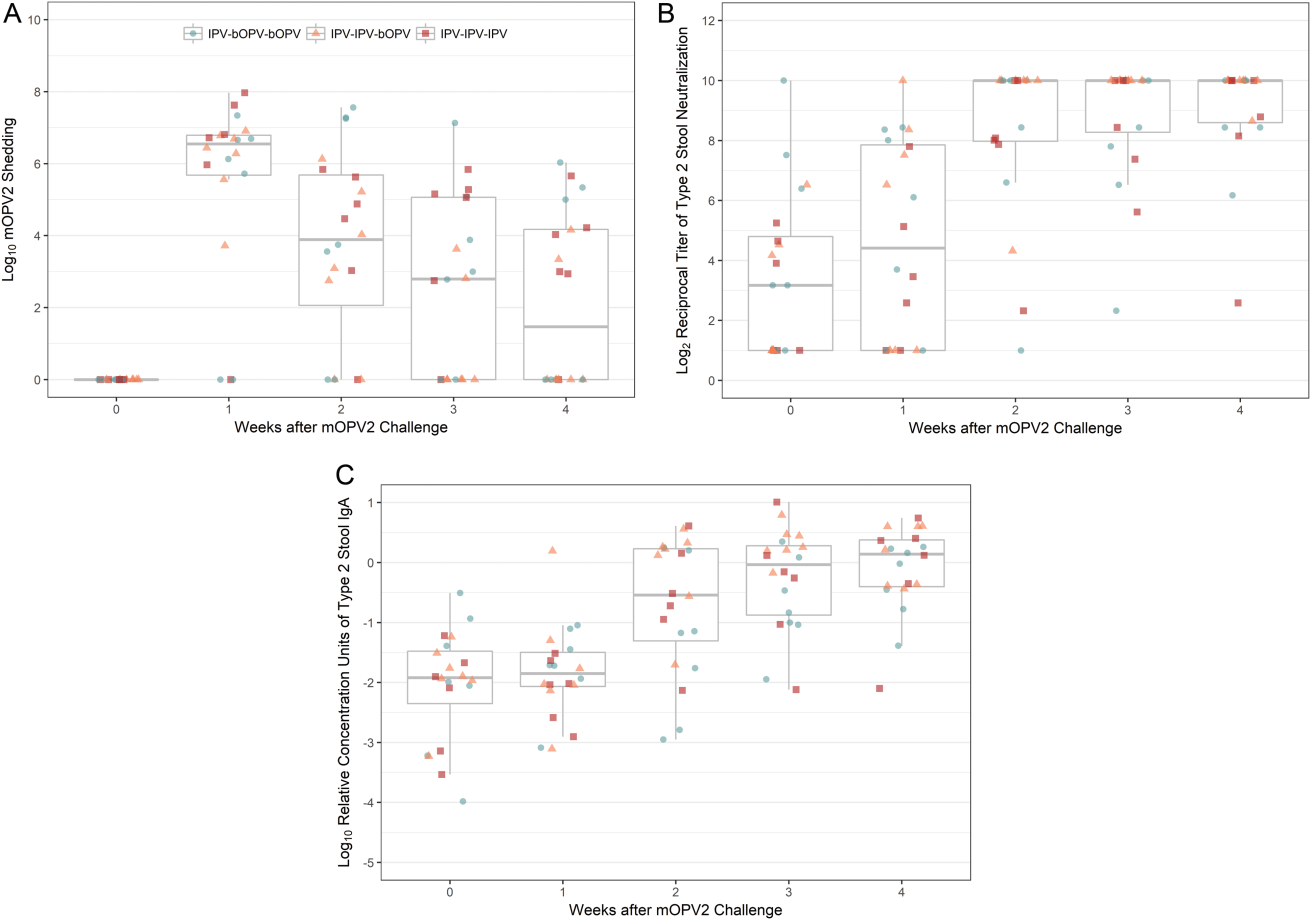
Polio type 2–specific intestinal immune responses to mOPV2 challenge in a random subgroup of infants (n = 20). (*A*) mOPV2 shedding, (*B*) type 2–specific stool neutralization, and (*C*) type 2–specific stool immunoglobulin A. Blue, circle-shaped markers indicate infants immunized with IPV-bOPV-bOPV (n = 7); orange, triangle-shaped markers indicate infants immunized with IPV-IPV-bOPV (n = 7); and red, square-shaped markers indicate infants immunized with IPV-IPV-IPV (n = 6). Abbreviations: bOPV, bivalent oral polio vaccine; IgA, immunoglobulin A; IPV, trivalent inactivated polio vaccine; mOPV2, monovalent oral polio vaccine type 2.

**Figure 4.**
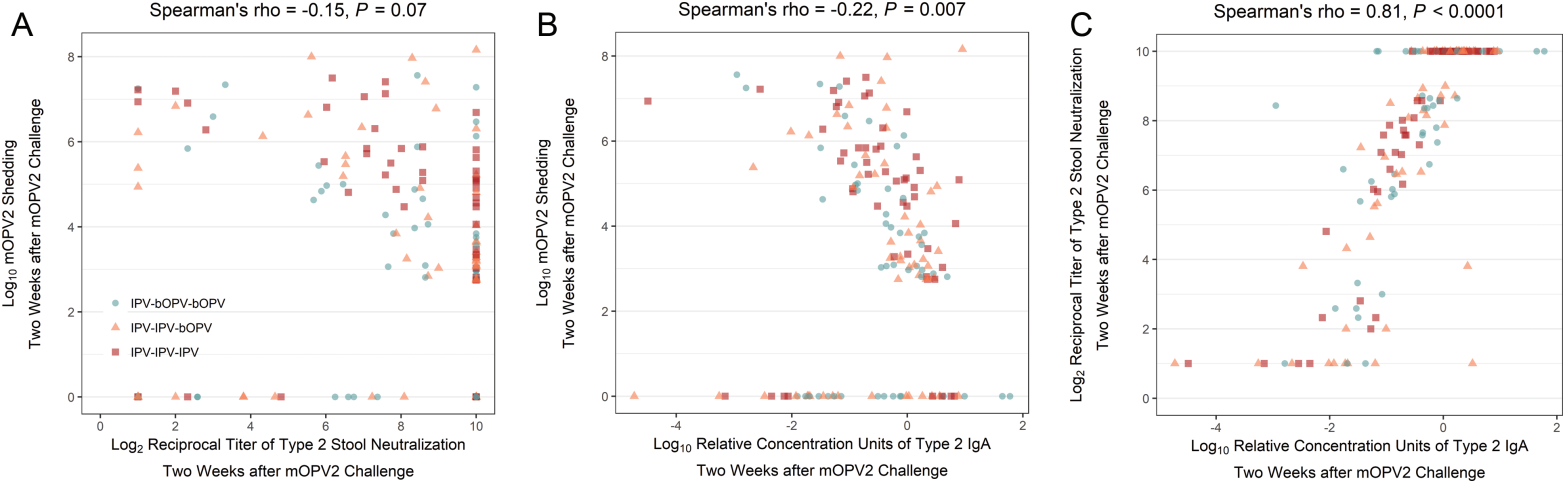
Pairwise correlations between type 2–specific stool neutralization, immunoglobulin A, and mOPV shedding 2 weeks after mOPV2 challenge (ie, 30 weeks of age). Abbreviations: bOPV, bivalent oral polio vaccine; IgA, immunoglobulin A; IPV, trivalent inactivated polio vaccine; mOPV2, monovalent oral polio vaccine type 2.

To investigate the extent to which the intestinal immunity induced by the IPV-bOPV and IPV-only immunization schedules has the capacity to provide protection upon subsequent exposure to live poliovirus, vaccinated infants were challenged with mOPV2 at 28 weeks of age. As previously reported, the magnitude of mOPV2 viral shedding peaked at 1 week after the challenge (Figure 3). At this time point, the mOPV2-derived virus was recovered in stool samples from 82% (n = 125/152) of participants, and the prevalence of shedders did not vary significantly by primary vaccine group assignment (*P* = .20, Chi-squared test). Viral shedding waned steadily over follow-up, and by 4 weeks post-challenge, less than half (47%, n = 71/152) of the participants continued to shed the virus. The polio type 2–specific mucosal responses appeared to closely track the viral replication patterns. Both type 2–specific stool neutralization and IgA concentrations began rising 1 week after the challenge (Figure 3) and became statistically significantly higher than baseline levels in all vaccine groups by 2 weeks post-challenge (Table 1). Levels of type 2–specific stool neutralization and IgA 2 weeks after the challenge were highly correlated (Spearman’s rho = 0.81, *P* < .0001) with each other and, to a lesser degree, both immune parameters were inversely correlated with mOPV2 shedding measured at the same point in time (stool neutralization: -0.15, *P* = .07; IgA: -0.22, *P* = .007; Figure 4).

**Table 1.** Poliovirus Types 1– and 2–Specific Serum and Intestinal Immune Responses to the Monovalent Oral Polio Vaccine Type 2 Challenge in Infants Previously Immunized With IPV-bOPV-bOPV, IPV-IPV-bOPV, and IPV-IPV-IPV

	Immune Marker	Weeks After Challenge	Weeks of Age	IPV-bOPV-bOPV (n = 48)		IPV-IPV-bOPV (n = 54)		IPV-IPV-IPV (n = 50)		*P* Value, Between Groups^b^
				Median (IQR)	*P* Value, Between Visits^a^	Median (IQR)	*P* Value, Between Visits^a^	Median (IQR)	*P* Value, Between Visits^a^	
Type 1	Serum Neutralization,	0	28	10.5 (10.5, 10.5)	*.11*	10.5 (10.5, 10.5)	*.10*	9.5 (8.8, 10.2)	*.0004*	*.0001*
	Log_2_ Reciprocal Titer	1	29	10.5 (10.5, 10.5)		10.5 (10.5, 10.5)		9.2 (8.5, 10.2)		*.0001*
	Stool Neutralization,	0	28	6.9 (5.3, 8.6)	*.45*	7.4 (3.8, 8.9)	*.58*	1.0 (1.0, 3.6)	*.41*	*.0001*
	Log_2_ Reciprocal Titer	2	30	7.4 (5.6, 8.9)		8.0 (3.9, 10)		1.0 (1.0, 3.5)		*.0001*
	Stool Immunoglobulin A,	0	28	-0.8 (-1.3, -0.6)	*.59*	-0.8 (-1.3, -0.5)	*.30*	-1.6 (-1.9, -1.2)	*.56*	*.0001*
	Log_10_ Relative Concentration Units	2	30	-0.9 (-1.1, -0.4)		-0.8 (-1.1, -0.5)		-1.5 (-1.9, -1.2)		*.0001*
Type 2	Serum Neutralization,	0	28	5.8 (3.8, 7.5)	*<.0001*	9.5 (7.2, 10.5)	*<.0001*	9.8 (9.2, 10.5)	*.0023*	*.0001*
	Log_2_ Reciprocal Titer	1	29	8.5 (5.2, 10.2)		10.5 (8.5, 10.5)		10.5 (9.5, 10.5)		*.0001*
	Stool Neutralization,	0	28	1.0 (1.0, 2.8)	*<.0001*	1.0 (1.0, 5.6)	*<.0001*	1.0 (1.0, 2.0)	*<.0001*	*.06*
	Log_2_ Reciprocal Titer	2	30	8.5 (6.1, 10)		8.6 (4.3, 10)		8.6 (6.6, 10)		*.74*
	Stool Immunoglobulin A,	0	28	-2.0 (-2.7, -1.5)	*<.0001*	-1.9 (-2.6, -1.6)	*<.0001*	-2.5 (-3.1, -2.0)	*<.0001*	*.004*
	Log_10_ Relative Concentration Units	2	30	-0.4 (-1.2, 0.1)		-0.3 (-1.2, 0.2)		-0.5 (-1.1, 0.2)		*.96*

Data shown are from NCT 01841671, undertaken in Santiago, Chile, between 25 April and 1 August 2013 (N = 152).

Abbreviations: bOPV, bivalent oral polio vaccine; IPV, trivalent inactivated polio vaccine; IQR, interquartile range.

^a^ *P* values are from Wilcoxon matched-pairs signed-ranks tests.

^b^ *P* values are from Kruskal-Wallis tests.

Of note, differences between the vaccine groups were observed in the IgA concentrations measured at the time of the challenge and in the overall shedding patterns, suggesting there may be some degree of cross-reactivity across polio serotypes. Despite not receiving a homologous live vaccine, infants in the IPV-bOPV groups had higher type 2–specific stool IgA levels at the time of the challenge than children immunized with IPV alone (Table 1; Figure 2). Further, in a linear regression adjusted for breastfeeding status at the time of the challenge, the shedding index endpoint decreased, on average, by 0.51 units (95% confidence interval: 0.18 to 0.85, *P* = .003) for each 1 standard deviation increase in the log_10_ type 2–specific stool IgA concentration measured at the time of the challenge. By 4 weeks after the challenge, only 39% (n = 40/102) of the infants in the 2 IPV-bOPV study arms continued to shed the virus, as opposed to 62% (n = 31/50) of the infants in the IPV-only group (*P* = .02, Chi-squared test). Also, supporting the hypothesis of heterotypic immunity, the percentage of children in the IPV-only group with detectable type 1–specific stool neutralization increased from 28% (n = 14/50) at the time of the challenge to 40% (n = 20/50) 2 weeks after receiving mOPV2.

### Potential Association Between Breastfeeding and Intestinal Immunity to Polio Type 2

**Figure 5.**
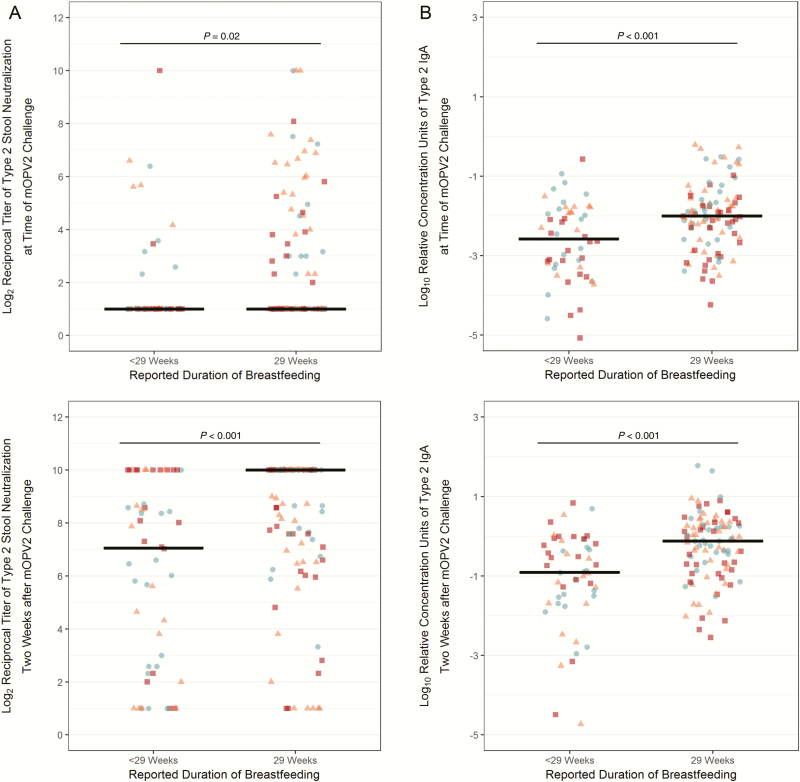
Intestinal immunity to polio type 2 at the time of and 2 weeks after monovalent oral polio vaccine type 2 challenge (ie, 28 and 30 weeks of age) by reported duration of breastfeeding. *P* values are from Mann-Whitney U tests comparing the distributions of immune markers by breastfeeding category. Horizontal black bars indicate the median levels. Blue, circle-shaped markers indicate infants immunized with IPV-bOPV-bOPV (n = 48); orange, triangle-shaped markers indicate infants immunized with IPV-IPV-bOPV (n = 54); and red, square-shaped markers indicate infants immunized with IPV-IPV-IPV (n = 50). Abbreviations: bOPV, bivalent oral polio vaccine; IgA, immunoglobulin A; IPV, trivalent inactivated polio vaccine; mOPV2, monovalent oral polio vaccine type 2.

In addition to vaccine group assignment, duration of breastfeeding was another variable that was associated with the levels of intestinal immune markers measured in stool samples. Participants whose mothers reported breastfeeding through 29 weeks of age (ie, up through and beyond the time of the mOPV2 challenge) had 2.5-fold higher odds (95% confidence interval: 1.1 to 5.4, *P* = .02, univariate logistic regression) of having detectable type 2 stool neutralization at the time of the challenge than their peers with a shorter reported duration of breastfeeding. Consistent with this observation, and relative to infants without concomitant breastfeeding, infants with reported breastfeeding at 29 weeks had significantly higher type 2 neutralization at 30 weeks of age (ie, 2 weeks post-challenge), as well as heightened stool concentrations of type 2–specific IgA at the 28 and 30 week visits (Figure 5). The shedding index endpoint was also lower, albeit not statistically significantly, in the children breastfed through 29 weeks (mean difference after adjusting for vaccine group in a multivariate linear regression: -0.61; 95% confidence interval, -1.28 to 0.06; *P* = .07).

## DISCUSSION

The substitution of the live type 2 component from oral polio vaccines for its inactivated counterpart represents a major step in the polio eradication endgame and serves as a programmatic precursor for the eventual cessation of all OPV use. However, along with introducing important opportunities for mitigating the risk of vaccine-associated paralytic poliomyelitis and the circulation of OPV2-derived strains, potential risks are associated with the withdrawal of live polio vaccines from immunization programs. Our findings indicate that, despite their demonstrated ability to elicit strong type 2 serum neutralization, IPV-bOPV and IPV-only immunization schedules induce limited type 2–specific intestinal neutralizing activity and IgA responses. Similarly, whereas robust type 1 serum immunity was induced regardless of vaccine group assignment, infants who received IPV alone were observed to have inferior type 1 intestinal immunity relative to their peers who had received at least 1 dose of bOPV as part of their primary vaccine schedule. When challenged with mOPV2, high levels of the mOPV2 virus were recovered from the stools of infants in each of the immunization groups, and almost half of all study participants continued to shed the virus 4 weeks after the challenge. Overall, these results substantiate concerns that populations with polio immunity driven by inactivated rather than live vaccines have the potential to act as conduits of transmission if OPV and/or wild poliovirus strains remain in circulation [19], such as was observed in the case of the 2013 silent outbreak of wild poliovirus type 1 in an IPV-vaccinated Israeli population [20, 21].

Although the relative protection afforded by polio vaccines is most commonly inferred from changes in serum antibody levels, a growing number of studies, 18 of which were meta-analyzed by Hird and Grassly [10], have used an OPV challenge model and its resultant viral shedding as a proxy measure of mucosal immunity. The current study advances our understanding of the OPV challenge system by directly measuring mucosal functional activity and antibody levels and defining their correlations with viral shedding. Furthermore, the present investigation of type 1–specific mucosal immunity to poliovirus provides proof of concept that, moving forward, it may be plausible to investigate vaccine-induced mucosal immunity to polio in the absence of an OPV challenge. In conjunction with the global withdrawal of OPVs, such an approach could be deemed necessary to eliminate the risks of introducing circulating vaccine-derived polioviruses to the environment. Moreover, a direct measurement of mucosal neutralizing activities and antibody responses may enable investigators to evaluate the intestinal immune impacts of novel vaccine candidates (eg, new, highly-attenuated, and genetically stable OPVs) in a dose-by-dose manner.

Broadly, our results, which found minimal intestinal immune responses even after 3 doses of IPV, align with earlier IPV-only shedding studies that have shown the odds of excreting a type 2 poliovirus after an OPV challenge are similar between unvaccinated children and those who received 2 [22] or 3 [12] doses of IPV. The findings are also consistent with shedding studies of IPV-bOPV mixed schedules, which have demonstrated that the addition of IPV to a primary bOPV schedule only marginally reduces the type 2 viral shedding index [18] and, indeed, that the odds of household transmission remain significantly higher in IPV-bOPV recipients relative to their tOPV-receiving counterparts [23]. Differing from these patterns, 4 studies in Indian children residing in regions with low OPV effectiveness [24] have provided evidence that a late, supplementary dose of IPV following multiple OPV immunizations can boost intestinal immunity for up to 11 months, as indicated by reduced poliovirus shedding following a bOPV challenge [25–27] and increased counts of serum antibody–secreting cells with mucosal homing markers [28]. Thus, the previous receipt of an OPV may prime children for IPV-induced intestinal mucosal responses that are comparable or better than responses to an additional dose of OPV.

In this study, approximately one-third of the cohort had detectable—albeit mostly low—type 2–specific stool neutralizing activity at the time of the challenge. The source of this underlying immunity remains poorly defined. As trivalent OPV was still being administered in the country at the time of the trial and as 3 infants were shedding prior to the challenge, some degree of passive exposure to Sabin-like viruses is probable. Another possibility is that the bOPV vaccine in the primary series could have had some degree of cross-reactivity, augmenting the type 2–specific IgA levels in the stool samples. Additionally, the subsidiary analyses provide intriguing, preliminary evidence that breastfeeding may be positively associated with mucosal immune indicators in the context of polio immunization, in line with several earlier studies of breastfeeding and serum neutralizing activity [29, 30]. Further research is needed to disentangle the relative contributions of breastfeeding-associated factors, such as the transfer of secreted IgA [31], programming of the gut microbiome [32, 33], and stimulation of infant intestinal IgA production, which could potentially underlie the observed enhancement of mucosal responses in concurrently breast-fed infants.

In sum, our study of Chilean infants’ mucosal immune responses to IPV-bOPV and IPV-only immunization schedules reinforces the concept that mucosal and systemic responses to polio vaccination are remarkably separate in their induction, functionality, and role in clinical protection against poliovirus and its transmission. Systemic (humoral) immunity is key to the prevention of paralytic poliomyelitis but, in the context of global eradication, mucosal immunity is equally essential through its role in halting intestinal viral shedding and, thereby, interrupting the fecal-oral transmission of both wild and vaccine-derived polioviruses. Indeed, if IPV does not substantively limit enteric viral replication on subsequent exposure to live polioviruses, children who live in conditions that favor fecal-oral transmission and those who have not received a vaccine with a live poliovirus type 2 component will potentially be at continued vulnerability to infection as long as OPV2-derived strains persist in the environment and/or could be re-introduced (eg, via laboratory [34] or manufacturing containment failures [35], or importation by immunocompromised long-term shedders of OPV2-derived viruses [36]).

## Supplementary Data

Supplementary materials are available at *Clinical Infectious Diseases* online. Consisting of data provided by the authors to benefit the reader, the posted materials are not copyedited and are the sole responsibility of the authors, so questions or comments should be addressed to the corresponding author.

## Supplementary Material

Supplemental_data

## Notes

P. F. W. conceptualized this study. The methodology was developed by M. E. A., M. A., and P. F. W. and the formal analysis and visualization by E. B. B. The investigation was conducted by W. W.-A., R. I. C., and A. W. B. with resources from W. C. W. and M. G. O. This manuscript was drafted by E. B. B., A. S. B., and P. F. W. and reviewed and edited by E. B. B., M. G. O., M. A., W. C. W., A. S. B., and P. F. W. Supervision was provided by A. S. B. and P. F. W. Funding was secured by P.F.W.

***Financial support.*** This work was supported by the Bill & Melinda Gates Foundation (OPP1104756 to P. F. W.).

***Supplement sponsorship.*** This article appears as part of the supplement “Polio endgame and beyond: Vaccine choices, transmission dynamics, and surveillance implications,” sponsored by The Bill & Melinda Gates Foundation.

***Potential conflicts of interest.*** A. S. B. is an employee at the Bill & Melinda Gates Foundation. All other authors report no potential conflicts. All authors have submitted the ICMJE Form for Disclosure of Potential Conflicts of Interest. Conflicts that the editors consider relevant to the content of the manuscript have been disclosed.
