# Supplementary material for: Intestinal Immunity to Poliovirus Following Sequential Trivalent Inactivated Polio Vaccine/Bivalent Oral Polio Vaccine and Trivalent Inactivated Polio Vaccine–only Immunization Schedules: Analysis of an Open-label, Randomized, Controlled Trial in Chilean Infants
Source: Clin Infect Dis. 2018 Oct 30;67(Suppl 1):S42–50. doi: 10.1093/cid/ciy603 (PMC6206105; doi:10.1093/cid/ciy603)
Supplement: Supplemental_data [file ciy603_suppl_supplemental_data.docx]

**Supplementary Figure 1.** Correlations of type 2-specific serum neutralization at the time of mOPV2 challenge (i.e., 28 weeks of age) with (A) Type 2-specific stool IgA at challenge and (B) mOPV2 viral shedding one week after challenge. Blue circle-shaped markers indicate infants immunized with IPV-bOPV-bOPV (N = 48), orange triangle-shaped markers indicate infants immunized with IPV-IPV-bOPV (N = 52), and red square-shaped markers indicate infants immunized with IPV-IPV-IPV (N=50). Abbreviations: IPV = trivalent inactivated polio vaccine; bOPV = bivalent oral polio vaccine; IgA = immunoglobulin A.

**A.**

**
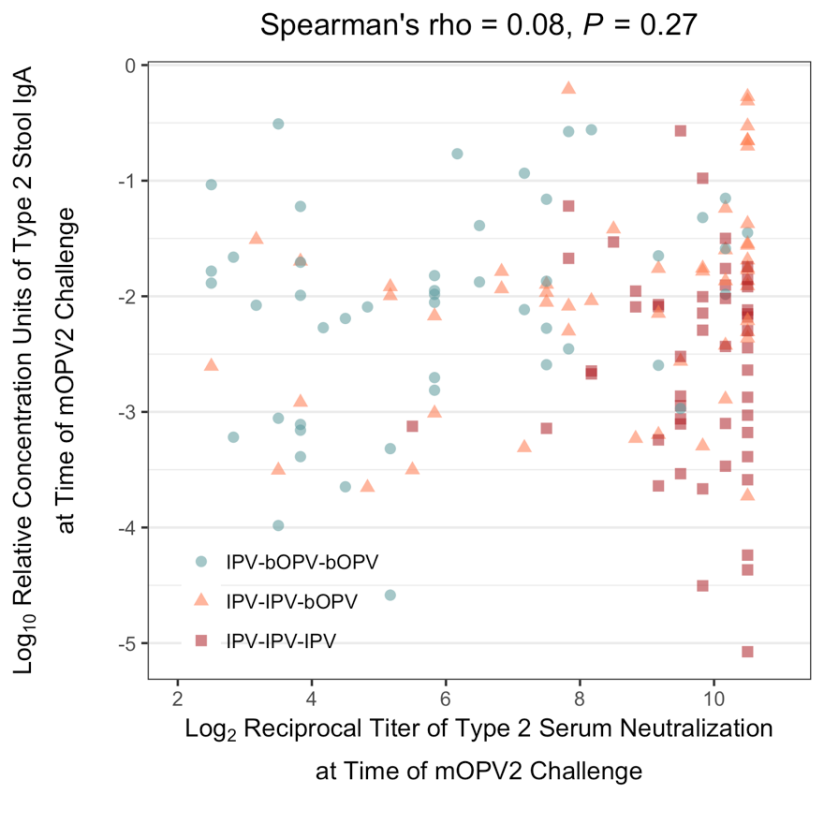
**

**B.**

**
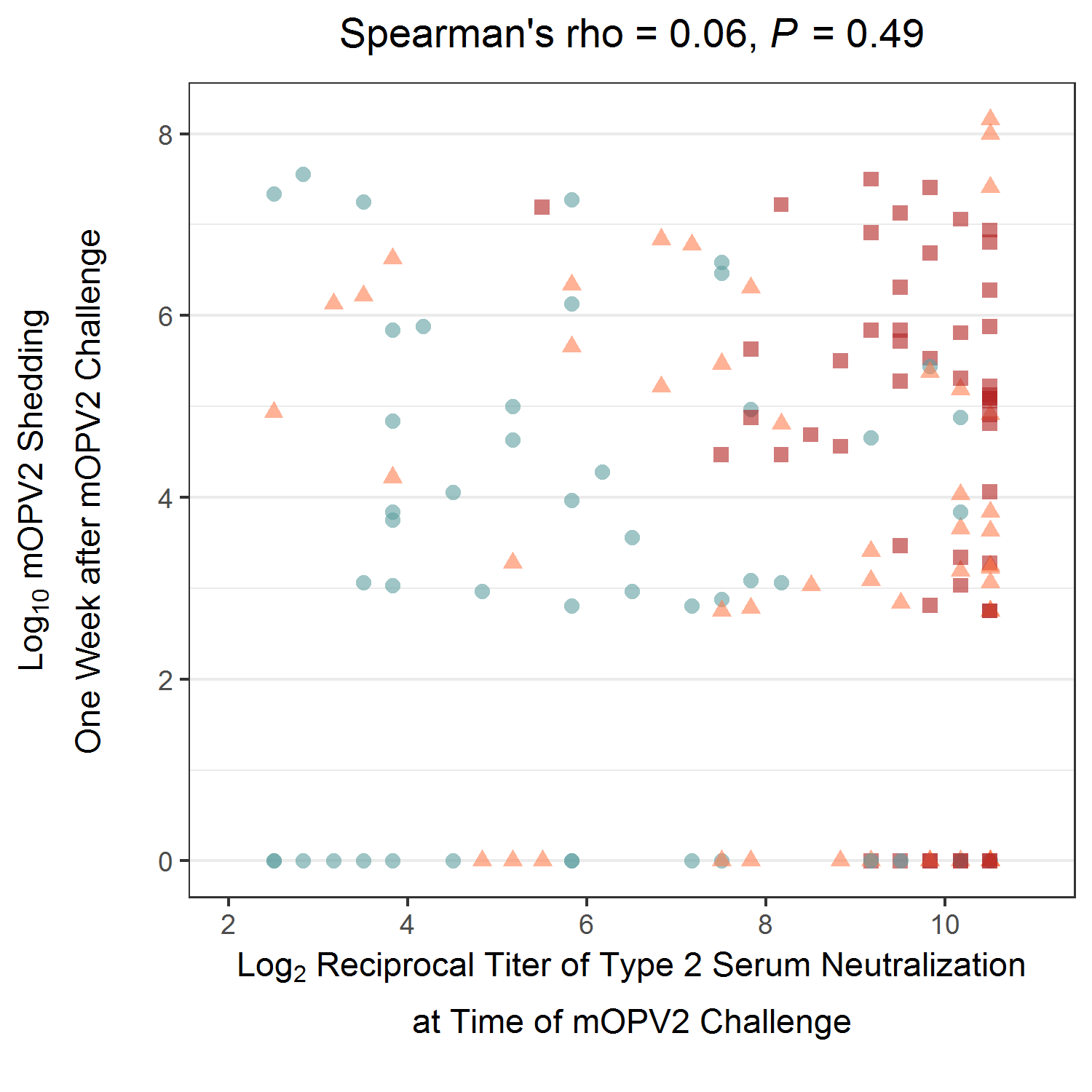
**
